# Validation of Freshly Isolated Rat Renal Cells as a Tool for Preclinical Assessment of Radiolabeled Receptor-Specific Peptide Uptake in the Kidney

**DOI:** 10.3390/ph16050696

**Published:** 2023-05-04

**Authors:** Pavel Barta, Petr Nachtigal, Jana Maixnerova, Lenka Zemankova, Frantisek Trejtnar

**Affiliations:** 1 Department of Biophysics and Physical Chemistry, Faculty of Pharmacy in Hradec Kralové, Charles University, Akademika Heyrovskeho 1203, 50005 Hradec Kralove, Czech Republic; 2 Department of Biological and Medical Sciences, Faculty of Pharmacy in Hradec Kralové, Charles University, Akademika Heyrovskeho 1203, 50005 Hradec Kralove, Czech Republic; 3 Department of Pharmacology and Toxicology, Faculty of Pharmacy in Hradec Kralové, Charles University, Akademika Heyrovskeho 1203, 50005 Hradec Kralove, Czech Republic; 4 Department of Cell Biology and Genetics, Faculty of Science, Palacky University, Slechtitelu 27, 78371 Olomouc, Czech Republic

**Keywords:** renal retention, radiotoxicity, nephrotoxicity, cellular model, radiopeptide, megalin

## Abstract

The synthetic analogs of regulatory peptides radiolabeled with adequate radionuclides are perspective tools in nuclear medicine. However, undesirable uptake and retention in the kidney limit their application. Specific in vitro methods are used to evaluate undesirable renal accumulation. Therefore, we investigated the usefulness of freshly isolated rat renal cells for evaluating renal cellular uptake of receptor-specific peptide analogs. Special attention was given to megalin as this transport system is an important contributor to the active renal uptake of the peptides. Freshly isolated renal cells were obtained from native rat kidneys by the collagenase method. Compounds with known accumulation in renal cells were used to verify the viability of cellular transport systems. Megalin expressions in isolated rat renal cells were compared to two other potential renal cell models by Western blotting. Specific tubular cell markers were used to confirm the presence of proximal tubular cells expressing megalin in isolated rat renal cell preparations by immunohistochemistry. Colocalization experiments on isolated rat kidney cells confirmed the presence of proximal tubular cells bearing megalin in preparations. The applicability of the method was tested by an accumulation study with several analogs of somatostatin and gastrin labeled with indium-111 or lutetium-177. Therefore, isolated rat renal cells may be an effective screening tool for in vitro analyses of renal uptake and comparative renal accumulation studies of radiolabeled peptides or other radiolabeled compounds with potential nephrotoxicity.

## 1. Introduction

The radiolabeled analogs of several regulatory peptides are perspective tools for scintigraphic radioimaging or for the radiotherapy of some malignancies [1,2]. Investigations have focused on radiolabeled somatostatin analogs and gastrin derivatives such as minigastrins (MG) [3,4]. Preclinical and clinical studies have evaluated compounds derived from octreotide or gastrin radiolabeled with various radionuclides and tested them as potential radiodiagnostic agents. Many analogical somatostatin or minigastrin analogs radiolabeled with beta-emitting radionuclides have been investigated as tools for treating patients with receptor-positive tumors [3,4]. However, the significant renal uptake of radiolabeled peptide analogs [5,6] may reduce the ability to detect small tumors in the perirenal region by scintigraphic imaging. It may also result in radiotoxicological injury to the kidney, limiting the peptides’ clinical use [7].

Several experimental models can be used to study renal transport, accumulation, and the retention of compounds in vitro. These models include isolated kidneys, precision-cut tissue, or cells. Various standard cell lines representing renal epithelial cells are particularly applicable to biomedical research. For example, OK (opossum kidney) cells, LLC-PK1 (porcine kidney) cells, MDCK (Madin–Darby canine kidney) cells, and HK2 (human kidney) cells are commercially available as renal in vitro models. OK cells and rat yolk sac epithelial cells have been used as cellular models in studies on the renal handling of radiolabeled peptides in vitro [8,9]. However, other cell types may also be viable for biomedical research. Such models may represent primary proximal tubular cells [10] or native renal cells freshly prepared from the animal kidney [11] immediately before transport studies. The established renal cell models differ in basic biochemical function parameters [12]; however, they also differ in the expression of drug transporters in the cellular membrane. Nevertheless, comparative studies on the differences in individual cell renal models’ membrane drug transporters are still scarce. Therefore, evaluations and comparisons of the usefulness of renal cellular models for particular groups of drugs and study types are necessary. In addition, other characteristics of the available renal cellular models should be investigated more thoroughly.

Renal accumulation is mediated by several transport mechanisms depending on many factors, such as molecular size, charge, and lipophilicity. Transporters localized at renal proximal tubular cell membranes, such as OATs (organic anion transporters) or OCTs (organic cation transporters), are responsible for the influx of organic anions or cations into renal cells with a relatively lower molecular size [13]. Larger peptides and proteins are transported from the urine by the multi-ligand endocytic megalin receptor complex localized at the apical membrane of the proximal renal tubules. Megalin (LRP2) is a large glycoprotein, a member of the low-density lipoprotein receptor family, and is abundantly expressed in the proximal kidney tubules and several other absorptive epithelia [14,15]. The megalin system mediates the transport of peptides and proteins such as albumin, plasminogen, polybasic drugs, or low-molecular-weight polypeptides such as insulin and angiotensin II [16]. This transport system plays an important role in the renal uptake of many radiopeptides that are potentially useful as radiodiagnostics or radiotherapeutics [5,7,9].

The present study examines the applicability of freshly isolated rat renal cells to transport and accumulation studies. It also considers the difficulties encountered in such studies. The value of primary renal cells for testing radiopeptide renal uptake and differences in megalin expression in several cellular renal models were evaluated. Several radiolabeled somatostatin and gastrin analogs labeled with indium-111 or lutetium-177 were employed as radiopeptide representatives. These compounds are known to accumulate to varying degrees in kidney tissue [5,6]. We used several radiolabeled compounds with known renal uptake to validate the transport functions of the prepared cells. Radiolabeled albumin and DMSA (dimercaptosuccinate) were selected as markers of proximal tubule endocytic activity. Both compounds are known as substrates of the megalin endocytic receptor [14,17]. [^99m^Tc]Tc-MAG3 (mercaptoacetylglycylglycylglycine) is transported into renal cells via an energy-dependent transport system for organic anions [18]. Therefore, its cellular uptake indicates the adequate energetic state of kidney cells and the integrity of cell membranes. To assess the method’s validity and compare our findings with previously published results, we compared megalin expression in cell models with native kidney tissue. We used immunochemistry to demonstrate proximal renal cells bearing megalin in experimental preparations of isolated rat renal cells.

## 2. Results

### 2.1. Megalin Expression Approvement

**Figure 1:**
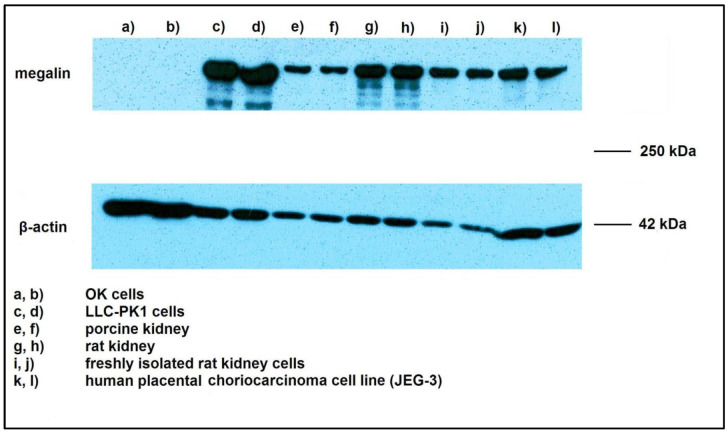
Western blot analysis of megalin expression in experimental renal cellular models of OK, LLC-PK1, and isolated rat cells compared to megalin expression in the porcine kidney, rat kidney, and human placental cells.

The Western blot analysis confirmed the expression of the megalin endocytic receptor in the studied cells and tissues. LLC-PK1 and isolated rat cells showed considerable expression, whereas the megalin detection in OK cells was unsuccessful. A strong expression was also found in the comparative preparations of porcine and rat kidney cells, as well as in JEG-3 human placental cells, which served as a positive control (Figure 1).

**Figure 2:**
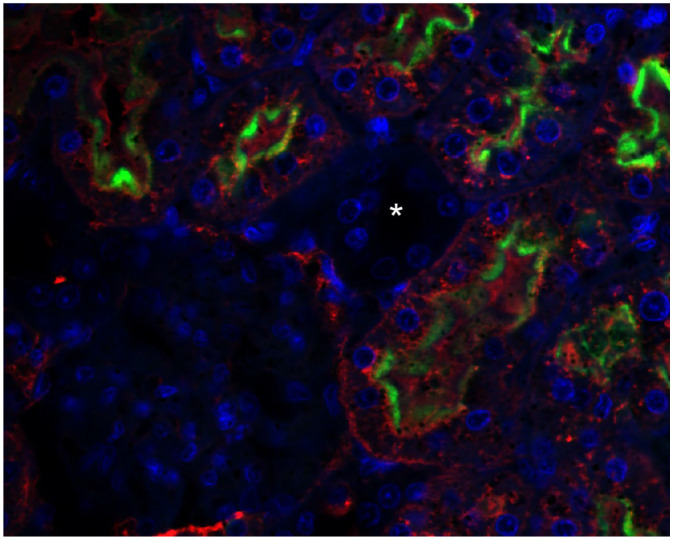
Colocalization of megalin and PHA-E in natural rat kidney tissue. Slight PHA-E staining is visible in the glomeruli. However, colocalization of megalin (green) and PHA-E (red) is observed only in the proximal tubules. By contrast, no staining is visible in the distal tubules (asterisk). DAPI was used for nuclei staining. Original magnification was 400×.

**Figure 3:**
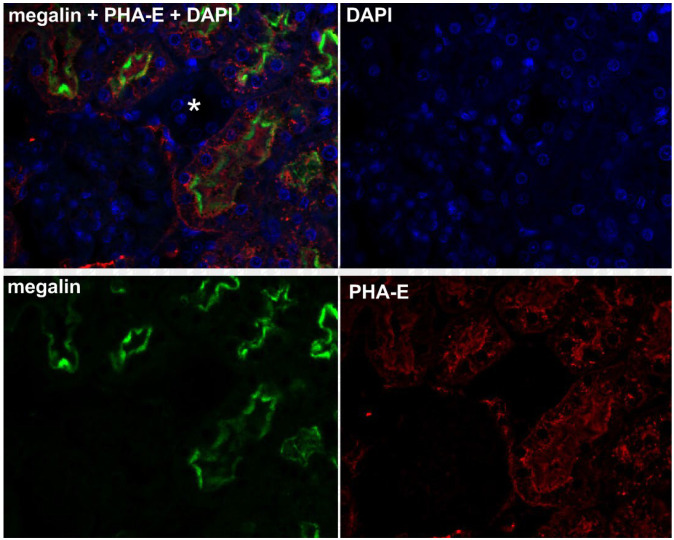
Colocalization of megalin and PHA-E in isolated rat kidney cells. Colocalization of megalin (green) and PHA-E (red) is evident in the proximal tubule cells (arrows). DAPI was used for nuclei staining. Original magnification was 400×.

**Figure 4:**
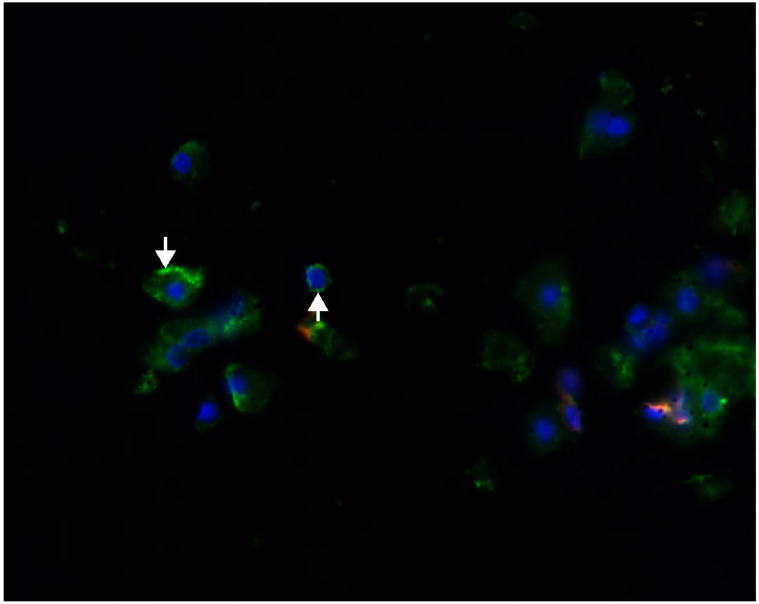
Colocalization of megalin and PNA in isolated rat kidney cells. No colocalization of megalin (green) and PNA (red) was detected in rat kidney cells. Megalin staining is visible only. Original magnification was 400×.

Fluorescence immunohistochemistry using specific markers of proximal tubules and distal tubules, PHA-E and PNA, confirmed the colocalization of megalin and PHA-E exclusively in the proximal tubules of the rat kidney (Figure 2). Moreover, the same colocalization was visible in isolated rat kidney cells (Figure 3). On the contrary, no colocalization of megalin or PNA in the distal tubules was detected in the rat kidney (Figure 4). The results of the immunohistochemical analysis clearly demonstrated megalin colocalization with proximal tubule markers (PHA-E) only, both in the rat kidney and rat kidney cells.

### 2.2. Evaluation of Cell Uptake In Vitro

**Figure 5:**
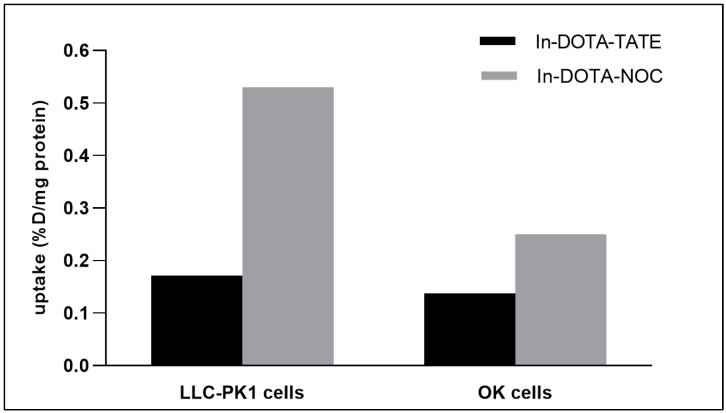
Accumulation of somatostatin receptor-specific analogs [^111^In]In-DOTA-TATE (In-DOTA-TATE) and [^111^In]In-DOTA-NOC (In-DOTA-NOC) in different renal cellular experimental models (incubation time = 30 min).

The cellular accumulation of two somatostatin receptor-specific analogs ([^111^In]In -DOTA-TATE and [^111^In]In -DOTA-NOC) was compared in two renal cellular models, OK cells and LLC-PK1 cells. Our results showed that cellular uptake following an incubation period of 30 min was relatively similar in both cell lines for [^111^In]In -DOTA-TATE (Figure 5). However, the [^111^In]In -DOTA-NOC accumulation observed in LLC-PK1 cells was significantly higher than that in OK cells (Figure 5).

Several radiolabeled peptides were incubated with isolated rat renal cells to compare their cellular retention. The results document different degrees of cell uptake (Table 1). The data on radiopeptide cellular uptake were compared with already available data on radiopeptide renal retention in rats in vivo, which were previously published by our team. The in vivo data show radioactivity in the kidneys 60 min after i.v. administration (Table 1). The tested somatostatin analogs generally exhibited a higher cell uptake in the isolated rat renal cells in comparison to the evaluated radiolabeled gastrin derivatives. [^111^In]In-DOTA-MG0 is the only exclusion among the tested minigastrins because its cellular accumulation is considerably high. The obtained data are in good accordance with previously published in vivo results. [^111^In]In-DOTA-MG0 retention in the rat kidney is relatively high, whereas renal retention of the other [^111^In]In-minigastrins is very low. Radiolabeled somatostatin analogs accumulate in the kidney in vivo relatively more intensively than most ^111^In-DOTA-minigastrins (Table 1).

**Figure 6:**
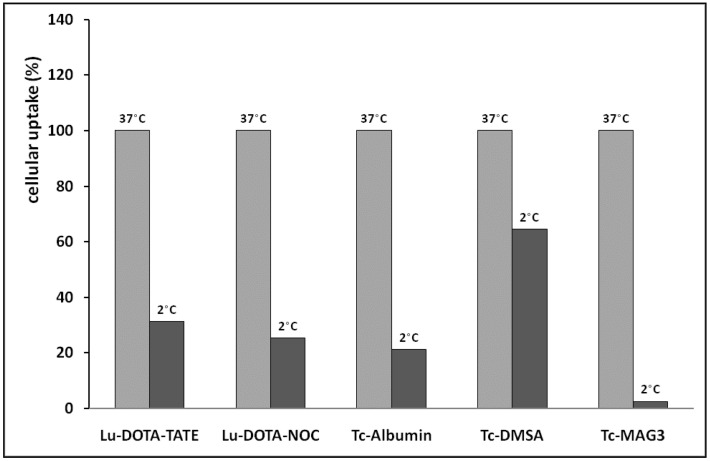
Comparative analysis of the active and passive cellular accumulation of selected radiolabeled compounds (*n* = 4) using rat-isolated renal cells. Accumulation at 37 °C is considered 100%. (Lu-DOTA-TATE = [^177^Lu]Lu-DOTA-TATE; Lu-DOTA-NOC = [^177^Lu]Lu-DOTA-NOC; Tc-albumin = [^99m^Tc]Tc-albumin; Tc-DMSA = [^99m^Tc]Tc-DMSA; Tc-MAG3 = [^99m^Tc]Tc-MAG3).

Another experiment tested renal cellular models’ usefulness in studying the active mechanisms of renal accumulation. The results presented in Figure 6 show that isolated rat renal cells can be used to compare the contribution of active membrane processes to the cellular accumulation of selected radiolabeled compounds. Generally, the obtained results confirmed the preservation of transport mechanisms in the isolated renal cells. The highest contribution of active accumulation to cell uptake was found in [^99m^Tc]Tc-MAG3. Only a negligible accumulation was observed in this compound at the low incubation temperature, which blocked active processes. The lowest contribution of active uptake to total accumulation was observed in [^99m^Tc]Tc-DMSA. The compound was accumulated predominantly by passive transport mechanisms. The tested radiolabeled peptides exhibited a relatively high participation of active transport processes in the total cell accumulation.

The experiment evaluating the influence of the cells’ growth period on the cellular uptake rate showed that [^99m^Tc]Tc-albumin accumulation by endocytosis was significantly lower in cells grown in a shorter time interval. Table 1 documents this finding for LLC-PK1 cells that grew for 6 h and for 3 days. A comparison of the uptake of ^99m^Tc-albumin in the two renal cell types revealed a significant difference in the cellular uptake between isolated rat renal cells and the LLC-PK1 cell line (Table 2).

## 3. Discussion

Cell lines are used extensively in biomedical research as in vitro models for kidney tissue. The validity of the data obtained in such experiments depends on the quality of the cell line, particularly when used as a surrogate for the tissue of origin. However, the typical parameters of cell lines may change due to various factors, and authentic characteristics may be lost [22]. Cell lines commonly used as in vitro models in biomedical studies can be unintentionally swapped, contaminated, or mutated. Any of these occurrences may result in different cellular parameter variations, including the amount of transport membrane proteins. In most cases, the cell lines lose transport expression to a significant extent. Drug transporters expressed in cell lines originating from relevant organs (e.g., kidney, liver, and intestine) may differ quantitatively and qualitatively from natural tissue [23,24]. Therefore, natural/primary cellular models should be considered for confirmation studies.

Freshly isolated cells may have advantages over renal cell lines; e.g., they exert similar functions to natural cells. In addition, according to a recently published interspecies comparison, rats appear to be the most suitable species for studying renal retention of radiolabeled peptides [6]. On the other hand, the complicated preparation and limited amount of cells available from one animal could be considered disadvantages of the method. Compared to conventional cell lines, performing the experiment is more demanding on the skill of the staff and the coordination of the experimental work. Another disadvantage of freshly isolated cells compared to standard cell lines is the limited lifespan of the cells. Therefore, the method is not suitable for studying the long-term accumulation of substances. Because the method includes the use of rat cells, interspecies differences should be considered. However, there is no relevant information on differences between rat and human megalin systems concerning peptide transport. The preparation of isolated renal cells contains a mixture of renal cell populations. However, this does not impede comparative studies on renal drug transport. In such cases, the renal preparation represents the kidney as a whole organ; thus, the accumulation could represent total renal uptake. It is necessary to use a separation method such as flow cytometry or centrifugation to identify which cell type is responsible for renal uptake. According to known data on transporter localization in the nephron [13], we can generally expect the most intensive drug uptake in renal proximal tubular cells.

Freshly isolated rat renal cells have been used in toxicological in vitro studies [25,26,27], physiological in vitro studies [28,29], metabolic phenotyping [30], and experimental studies on drug metabolism [31,32]. Although experimental studies on peptide accumulation in the kidney may be successfully performed in vitro using selected cell lines, such as OK (opossum kidney) cells [8], isolated rat renal cells might also help analyze the renal handling of xenobiotics, including radiolabeled compounds [33]. This method may apply to radiolabeled peptides that are potentially useful in radiodiagnosis and targeted radiotherapy, such as somatostatin analogs, since they are, in most cases, excreted from the body via the kidney [5,9]. Therefore, radiopeptide concentrations in the urine and kidney may be relatively high. This pharmacokinetic characteristic is the basis for undesirable renal uptake and the retention of radiolabeled peptides. Furthermore, radioactive accumulation in the patient’s kidney may lead to the radiotoxic damage of the renal tissue [9,34].

To assess the mechanism and renal uptake rate of the model peptides, comparing their accumulation rate to the uptake of compounds with known renal accumulation and transport mechanisms into cells is useful. For example, albumin may serve as a reliable marker of active endocytosis. Albumin is intensively reabsorbed in the proximal renal tubules and transported into renal cells by megalin-mediated endocytosis. Therefore, the compound is a common substrate of this endocytic receptor [35,36]. Similarly, [^99m^Tc]Tc-DMSA accumulates in the kidneys via megalin-mediated endocytosis [17]. The renal accumulation of [^99m^Tc]Tc-DMSA significantly depends on retaining megalin receptor function. Therefore, it can serve as a marker of proximal tubule endocytic activity. Highly accumulated experimental compounds can be compared quantitatively with [^99m^Tc]Tc-MAG3 renal uptake. This agent is a compound that accumulates intensively in renal tissue and cells via an active transport system for organic anions [18].

Accumulation studies with several radiolabeled peptides were conducted to compare the usefulness of isolated rat renal cells for in vitro transport studies. We used both this model and selected standard renal cell lines. The obtained experimental data suggest different renal uptake rates in individual renal cellular models. LLC-PK1 cells showed similar uptake in one radiopeptide but significantly different uptake in another. In addition, the ^99m^Tc-albumin active uptake was significantly higher in isolated rat renal cells than in LLC-PK1 cells. Such experimental data document that several renal cellular models may be advisable for renal transport and receptor-specific radiopeptide accumulation studies. The relatively high retention of ^99m^Tc-MAG3 and ^99m^Tc-albumin in isolated rat cells confirms the cells’ good metabolic state and maintenance of transport function throughout the time interval used.

One problem with using in vitro cellular models is the variable expression of cellular components during passaging and growing. Variations in CYP450 isoenzyme activity in cell lines used to study biotransformation processes have been well described [37]. Similarly, drug membrane transporters may be expressed differently in cell lines depending on the origin or culture conditions [38,39]. However, such data are very scarce. We demonstrated that, in the case of radiopeptides, in vitro accumulation might be affected by the interval between seeding the cells and the time of the accumulation experiment. This finding means that the transport rate and cellular accumulation may depend on the cell growth phase. Although the time interval for observing differences in transport activity was much longer than for the cell lines, age-related changes in the endocytic receptors megalin and cubilin have been described in rats in vivo [40]. However, this will have only minor significance if experiments with cell lines are conducted under a constant regime and time intervals concerning seeding.

Based on the abovementioned variations in transport system activity, it is important to assess the expression of the studied transporter/s in the cellular model being used. We used Western blotting to analyze megalin expression in renal cellular models. Moreover, we compared megalin expression in both cells and kidney tissue. This analysis confirmed the presence of megalin endocytic receptors in the studied cells and tissues. LLC-PK1 cells and isolated rat renal cells showed considerable expression, whereas megalin detection in OK cells was unsuccessful. Strong expression was also found in comparative porcine and rat kidney preparations and in JEG-3 human placental cells. Such findings align with known data showing that the placenta and the tubular renal epithelium are two locations exhibiting significant megalin expression [36,41].

In our experiment, the most probable explanation for our inability to confirm megalin expression in OK cells was the specificity of the anti-megalin antibody used. The antibody is intended for use in human and rat tissue; thus, it reacts with human and rat megalin but not with opossum kidney protein. Since an anti-megalin antibody for opossum use is unavailable, commercially available opossum kidney cells present a relative disadvantage compared to rat and human renal cells regarding megalin detection by blotting.

The results of the immunohistochemical analysis in isolated rat kidney cells demonstrated megalin colocalization using biochemical markers in the proximal tubular cells. These indicators are found specifically in the proximal cell membrane. We did not see any significant megalin colocalization using markers in distal tubular cells. Megalin, the receptor responsible for active endocytosis, was expressed in both isolated rat renal cells and LLC-PK1 cells. These findings agree with previous reports on megalin localization in renal tissue and renal epithelium [36,42].

The presented study is focused on a possible application of the method using isolated rat renal cells in preclinical investigation on new peptide radiopharmaceuticals. Although other structural groups of potential radiopharmaceuticals may also exert an undesirable retention in the kidney [43,44], the renal accumulation of radiopeptides is a particularly interesting research area. Because of the risk of radiotoxicological kidney damage, special precautions must be taken in clinical practice in patients treated with radiolabeled peptides such as somatostatin analogs, which include an infusion of selected amino acids or other protective measures [45]. Moreover, the number of potential radiolabeled receptor-specific peptides under investigation is increasing. The group includes many peptide subgroups focused on various receptor targets. However, the undesirable renal uptake is solved by preclinical studies in many types of radiopeptides [46,47]. Therefore, the renal retention of radiopeptides using a wider range of methods remains an important research issue.

## 4. Materials and Methods

### 4.1. Western Blot Analysis

Megalin expression by Western blotting was studied in several types of renal cells (OK cells, LLC-PK1 cells, and freshly isolated rat renal cells) and compared to expressions in rat and porcine native renal tissue and a human placental cell line (JEG-3 cells) that is known to express megalin [48].

The excised rat and porcine kidney tissues were homogenized using Ultra Turrax Tube Drive (IKA, Staufen, Germany) with 10 mmol/L of Tris-HCl, 250 mmol/L of sucrose, 1 mmol/L of EDTA with pH 7.6, 0.5 µg/mL of leupeptin, 2 µg/mL of aprotinin, 50 µg/mL of benzamidine, and 40 µg/mL of phenylmethylsulfonyl fluoride (PMSF). The homogenate was centrifuged at 10,000× *g* for 60 min at 4 °C [49,50]. The LLC-PK1 and OK cells were grown in a 75 cm^2^ tissue culture flask and were lysed using a cell lysis buffer containing 0.5% of SDS and 10 mmol/l of Tris/HCl, pH 7.4. It was supplemented with 0.5 µg/mL of leupeptin, 2 µg/mL of aprotinin, 50 µg/mL of benzamidine, and 40 µg/mL of PMSF. The homogenate was centrifuged at 3000 g for 10 min at 4 °C [51,52]. The BCA (bicinchoninic acid) method determined the protein content of the resulting supernatants [53].

A Power Pac HC (BioRad, UK) was used to separate protein extracts for megalin by sodium dodecyl sulfate-polyacrylamide gel electrophoresis (SDS-PAGE) on a 6.25% gel (9 µg protein/lane). The proteins were electrotransferred onto a polyvinylidene (PVDF) membrane (Sigma–Aldrich, St. Louis, MO, USA). The protein transfer was checked routinely by staining the membrane with Ponceau S solution (Serva, Heidelberg, Germany). For immunoblotting, the antibodies were dissolved in 5% low-fat dried milk (BioRad, Hercules, CA, USA) in a Tris-buffered saline solution (TBS) containing 0.05% Tween (Duchefa Biochemie, Haarlem, The Netherlands). The membrane was immunoblotted with rabbit anti-megalin (donated by Prof. D. Biemesderfer, Yale University School of Medicine, New Haven, CT, USA) and mouse anti-β-actin antibodies at dilutions of 1:8000 and 1:5000, respectively. Megalin detection was performed with the appropriate horseradish peroxidase (HRP)-conjugated secondary antibodies at 1:2000 dilution (GE Healthcare, Amersham, UK) and 1:8000 for β-actin (Sigma–Aldrich, St. Louis, MO, USA) and enhanced with a chemiluminescence reagent (Thermo Scientific, Waltham, MA, USA).

### 4.2. Immunohistochemistry

*Phaseolus vulgaris Erythroagglutinin* (PHA-E) lectin was used as a specific marker of proximal tubulus, and *Peanut Agglutinin* (PNA) was used as a specific marker of distal tubules as previously published [54,55].

Rat kidneys were fixed by immersion in 4% neutral formaldehyde for 3 days and embedded in paraffin, after which 6 μm thick sections were cut. The renal rat cells were fixed in a 4% neutral formaldehyde solution for 1 h. They were immersed in OCT (optimal cutting temperature) embedding medium (Leica, Prague, Czech Republic) and then frozen in a freezer and stored at −20 °C. Afterward, serial cross-sections (7 μm) were cut on a cryostat and placed on gelatin-coated slides.

For the double fluorescence staining, we used a goat anti-rabbit secondary antibody marked with a green fluorochrome (CY2) (Jackson Immunoresearch, Suffolk, UK) (diluted at 1/100 in 5% bovine serum albumin (BSA) in phosphate-buffered saline (PBS)) to detect a rabbit anti-megalin antibody (anti-MC-220) at 1/100 dilution in 5% BSA in PBS for 1 h at room temperature. ExtrAvidin^®^−Cy3™ (Sigma–Aldrich, St. Louis, MO, USA) diluted at 1/500 in 5% BSA in PBS was used to detect either biotinylated Phaseolus Vulgaris Erythroagglutinin (PHA-E–diluted at 1/500 in 5% BSA in PBS; Vector Laboratories, Newark, CA, USA) or biotinylated Peanut Agglutinin (PNA—diluted at 1/500 in 5% BSA in PBS; Vector Laboratories, Newark, CA, USA) in the rat kidney and rat kidney cells. 4′,6-diamidino-2-phenylindole dihydrochloride (DAPI) (Sigma–Aldrich, St. Louis, MO, USA) was used for nuclei staining.

Microscopic photo documentation and image digitizing were performed using the Olympus AX 70 light and fluorescence microscope. We used a VDS Vosskuehler CD-1300QB monochromatic camera for fluorescence along with image analysis software NIS (Laboratory Imaging, Prague, Czech Republic).

### 4.3. Experiments with the Isolated Rat Renal Cells

Fresh rat renal cells were isolated from the kidney using the two-phase collagenase perfusion method described by Jones et al. [11]. Male Wistar rats weighing 270–330 g were used for the experiments. The rats were housed under standard conditions (tap water, standard diet, and a 12/12 h light–dark cycle). Animals were fasted for 18–24 h prior to experiments. The animal experiments were approved by the Ethical Committee of the Ministry of Education, Czech Republic (approval no. MSMT-7041/2014-11), and complied with Czech laws concerning animal protection.

As a first step, rats were anesthetized (sodium pentobarbital, 50 mg/kg), the abdomen was opened, and the right renal artery and adjacent parts of the aorta were freed. Then, the right renal artery was cannulated via the aorta. Single-pass perfusion was conducted using oxygenated calcium-free Hanks’ buffer (pH 7.4) containing EGTA (0.5 mM) and Hepes (25 mM). The second perfusion phase was performed in a recirculation system with modified Hanks’ buffer containing CaCl_2_ (4 mM) and collagenase (0.10 % *w*/*v*) for 18–22 min. The kidneys were removed and dispersed in cold Krebs–Henseleit buffer with Hepes (25 mM) and bovine serum albumin (2% *w*/*v*). The resulting suspension was filtered and washed with Krebs–Henseleit buffer. Following centrifugation (3 min; 80 g; 4 °C), we used the trypan blue exclusion method to determine the number of cells in the suspension. The preparations used had an initial viability of 88–93%. As a result, the cell viability at the end of the 30 min incubation interval was >85%.

Standard incubations were conducted for 30 min at 37 °C using 1 mL of cell suspension containing 2.10^6^/mL renal cells. The radiopeptide uptake was determined by mixing 1 mL of cell suspension and 10 µL of the radiopeptide (1 µg/mL). After incubation, the cells were washed four times and separated. The radioactivity of the cell fractions was measured as mentioned below.

An experiment was arranged to test this cellular model’s potential for investigating active membrane transport processes in radiopeptide renal accumulation. The accumulation rates of [^111^In]In-DOTA-TATE and [^111^In]In-DOTA-NOC in isolated renal cells were compared to several compounds with known uptake in renal tubular cells, namely, ([^99m^Tc]Tc-albumin, [^99m^Tc]Tc-MAG3, and [^99m^Tc]Tc-DMSA). Using the same procedure as for the radiopeptides, we tested and expressed the uptake of the other studied compounds. In addition to incubations at 37 °C, experiments at a low incubation temperature (2–4 °C) were performed to compare the role of active transport processes in the renal uptake of the studied agents.

### 4.4. Radiolabeling of Peptides

[DOTA^0^, D-Phe^1^, 1-Nal^3^]-octreotide (DOTA-NOC), [DOTA^0^, Tyr^3^, Thr^8^]-octreotide (DOTA-TATE), minigastrins (MG) DTPA-MG0 (DTPA-*D*Glu-Glu-Glu-Glu-Glu-Glu-Ala-Tyr-Gly-Trp-Met-Asp-Phe-NH_2_), DOTA-MG11 (DOTA-*D*Glu-Ala-Tyr-Gly-Trp-Met-Asp-Phe-NH_2_), DOTA-MG11 (DOTA-*D*Glu-Ala-Tyr-Gly-Trp-Met-Asp-Phe-NH_2_), DOTA-MG45 (DOTA-*D*Gln_6_-Ala-Tyr-Gly-Trp-Nle-Asp-Phe-NH_2_), and DOTA-MG47 (DOTA-*D*Gln_6_-Ala-Tyr-Gly-Trp-Met-Asp-Phe-NH_2_) were purchased from piCHEM (Graz, Austria). ^111^InCl_3_ and ^111^LuCl_3_ were purchased from GE Healthcare Ltd. (Amersham, UK) and Perkin Elmer (Boston, MA, USA), respectively. MAG3 (mercaptoacetylglycylglycylglycine) and DMSA (dimercaptosuccinate) were obtained from the Nuclear Research Institute (Rez, Czech Republic).

We prepared DOTA-TATE and DOTA-NOC labeled with ^111^In using the following method: An amount of 200 μL of 0.4 M acetate buffer (pH 5) with 0.24 M of gentisic acid, 10 μg of peptide in 10 μL of H_2_O, and 19 MBq of ^111^InCl_3_ in 0.04 M HCl were added to a vial. The solution was mixed thoroughly and incubated at 90–95 °C for 25 min. DOTA-TATE and DOTA-NOC labeled with ^177^Lu were prepared using 30 µL of 0.4 M acetate buffer (pH 5), 3 µg of peptide in 3 µL of H_2_O, and 46 MBq of ^177^LuCl_3_ in 0.05 M HCl. The solution was mixed thoroughly and incubated at 92 °C for 30 min. For somatostatin peptide conjugates, labeling efficiency was determined by instant thin-layer chromatography using 0.1 M of ammonium acetate with 10 mM of EDTA as mobile phase 1 and 10% ammonium hydroxide with methanol as mobile phase 2 (ratio 1:9). Radiochemical purity analysis using HPLC was performed on a column of LiChroCART 125–4 HPLC Cartridge Purospher RP18e at 5 mm (Merck, Darmstadt, Germany) using a Pharmacia LKB system linked to a Gradient Master GP 962 (Institute of Organic Chemistry and Biochemistry, Prague, Czech Republic) and operated with a UV detector and radioactivity monitoring analyzer. Mobile phases A (0.1% TFA in water) and B (CH_3_CN) were used with the following gradients: 0–5 min 0% B; 5–25 min 0–30% B; 25–30 min 30% B; 30–35 min 30–100% B; 35–40 min 100% B. The flow rate was maintained at 0.5 mL/min. [^99m^Tc]Tc-MAG3 and [^99m^Tc]Tc-DMSA were prepared and assessed according to the manufacturer’s instructions. The [^99m^Tc]Tc-albumin was prepared according to Dekker et al.’s method [56]. A Wallac 1480 Wizard 3” gamma-counter (Wallac, Turku, Finland) measured ^111^In, ^177^Lu, and ^99m^Tc activity in the biological samples.

Radiolabeling of minigastrins with ^111^In was performed by adding about 19 MBq of ^111^InCl_3_ in 0.5–1 μL of 50 mM HCl to 50 μL 0.4 M sodium acetate buffer (pH 4.5) with 10 μg of the peptide. After incubating at 80 °C for 30 min, the quality control of the product was determined by a gradient HPLC analysis [21].

A purification step following radiolabeling was not used because the employed procedures resulted in high radiolabeling yields. Radiochemical purity of the radiopeptide preparations was in the range of 97–99%. Specific activity of ^111^In- and ^177^Lu-labeled peptide preparations ranged from 1.5–2.5 to 12–15 MBq/µg, respectively. For biological experiments, all radiopeptide solutions were diluted with saline to a concentration of 1 μg/mL.

## 5. Conclusions

The results of the present study and previous studies suggest that, along with other cellular renal models, isolated rat renal cells might serve as a helpful tool for comparative transport studies and analyses of renal transport mechanisms in radiolabeled peptides and other investigational radiopharmaceuticals. The presented experimental in vitro model can be used not only to quantitative determination of potential renal uptake but also to analyze the contribution of active and passive transport to renal accumulation. The preservation of the main renal transport mechanism for the endocytic uptake of peptides and large proteins, megalin, can be considered an important advantage of the model. Future applications of the method could also include the experimental testing of renal retention in high molecular weight substances with considerable scientific significance such as radiolabeled antibody fragments or aptamers. The established method is also applicable for studies of the renal accumulation of non-radioactive drugs, nanoparticles, and other new drug carriers. Due to certain limitations, combinations with other available cell or organ models may improve the translational potential of the obtained data.

## Figures and Tables

**Table 1:** Comparison of radiopeptide uptake in isolated rat cells (%D/10^6^ cells) and in rat kidneys in vivo (%D/g).

Radiopeptide	In Vitro	In Vivo
[^111^In]In-DOTA-TATE	0.36 ± 0.10	3.08 ± 0.42 [19]
[^111^In]In-DOTA-NOC	0.75 ± 0.09	2.84 ± 0.28 [19]
[^111^In]In-DTPA-MG0	2.52 ± 0.58	26.4 ± 14.6 [20]
[^111^In]In-DOTA-MG11	0.16 ± 0.15	0.84 ± 0.35 [21]
[^111^In]In-DOTA-MG45	0.26 ± 0.16	1.53 ± 1.35 [21]
[^111^In]In-DOTA-MG47	0.30 ± 0.03	1.84 ± 1.40 [21]

**Table 2:** Comparison of [^99m^Tc]Tc-albumin uptake in young (grown for 6 h) and older (grown for 3 days) LLC-PK1 cells and isolated rat renal cells (mean ± S.D.; *n* = 4).

	Young LLC-PK1 Cells	Older LLC-PK1 Cells	Rat Renal Cells
Cellular uptake (%D/10^6^ cells/30 min)	0.036 ± 0.006	0.083 ± 0.015 ^★^	2.188 ± 0.411 ^§^

^★^ Statistically significant differences in younger LLC-PK1 cells at *p* < 0.05; ^§^ significant differences in older LCC-PK1 cells at *p* < 0.05.

## Data Availability

Not applicable.
